# Semaphor Strain

**Published:** 1915-08

**Authors:** 


### Semaphor Strain. A New Occupational Condition and Its Effects.

It is a well known fact that occupations, such as those of plastering and painting, in which the arms are much used in a state of abduction, are particularly trying. Partly on account of the relatively slight demand for raising the arms, partly on account of the tension on branches of the brachial plexus, abduction of the upper arms induces a degree of fatigue out of proportion to the actual muscular work done, while holding the arms outstretched for more than a minute or so is a particularly trying exercise. In more or less modified form, it held a place in the ancient system of torture.

A few years ago, street crossing service was regarded as a sinecure for policemen. In the present days of automobile traffic, not only has the name of this service been changed but it has become a work of great responsibility, requiring constant attention, quick judgment, much self control, and involving some degree of danger. By turning the traffic policeman into a human semaphor, he is subjected to the muscular strain mentioned with its attending liabilities to myalgia and even neuritis, to physical and mental fatigue while his station in the center of a street crossing involves an undue degree of exposure to weather which may react locally upon the especially strained tissues or in ordinary general ways. A minor but not entirely negligible factor in the case of a man on a relatively small salary and trained to habits of neatness, is the fact that dust, mud, oil and grazing by vehicles make the expense and trouble of maintaining a uniform greater for the traffic police than for those in any other branch of the service. Thus, with the exception of especially dangerous and difficult details, the traffic service is now considered the hardest duty of the police department.

We, therefore, advocate the adoption of mechanic semaphors which can be operated by an officer, either stationed at the corner of streets within the curb line or in an elevated box, over the center of the street crossing, whence an even better view can be had. We advocate this change not simply in the interests of the police officers but because mechanic semaphors are more visible—the policeman often being hidden by passing street cars or other vehicles—and because the fatigue and often the actual pain of acting as a human semaphor distracts the attention of the officers from the observation of traffic and renders him incapable of performing his executive duties with the instantaneous promptness and disinterested judgment so much needed.

General principles of efficiency are so well understood that, in no private business, would a man be required to do the work which could be so much better performed by a mechanic device nor would any intelligent manager of a private business require tact, courtesy, alertness and executive ability in an emergency and then hamper the discharge of duty in such a position by imposing unnecessary physical strain, pain and fatigue.

